# Barriers to referral uptake from primary to secondary eye care in the L V Prasad Eye Institute Network in South India

**DOI:** 10.1371/journal.pone.0325974

**Published:** 2025-06-26

**Authors:** Debananda Padhy, Giridhar Pyda, Rohit C. Khanna

**Affiliations:** 1 Allen Foster Community Eye Health Research Centre, Gullapalli Pratibha Rao. International Centre for Advancement of Rural Eye care, L V Prasad Eye Institute, Hyderabad, India; 2 Brien Holden Eye Research Centre, L.V. Prasad Eye Institute, Hyderabad, India; 3 School of Optometry and Vision Science, University of New South Wales, Sydney, Australia; 4 University of Rochester, School of Medicine and Dentistry, Rochester, New York, United States of America; King Khaled Eye Specialist Hospital & Research Centre, SAUDI ARABIA

## Abstract

**Purpose:**

To investigate the barriers preventing referral uptake from primary care vision centres (VC) to higher level secondary centres (SC) in rural South India.

**Methodology:**

This is a cross-sectional study conducted using data from 10 VCs surrounding an SC in Thoodukurthy village, Mahabubnagar district, Telangana, India. The study included 2,508 participants who received primary eye care at the VCs and referred to SC between July 1st to December 31st, 2019, and from July 1st to December 31st, 2020 respectively. Of these,1930 (76.9%) participants were available for the study. Participants were categorized as compliant if they visited the SC within one year of referral. Non-compliance was defined as failing to do so within that time. Interviews were conducted with non-compliant participants to understand their reasons for not seeking further care.

**Results:**

Among the 1930 participants 1507 (78%) were interviewed; 938 (62.2%) were compliant, and 569 (37.8%) were non-compliant. The mean age was 54.64 years (SD:14.28 years) and 716 (47.5%) were female. Multivariable analysis showed participants not referred for teleophthalmology (OR:1.41,95%CI:1.00–1.99), unmarried participants (OR:1.32,95%CI:1.02–1.71) and participants without formal education (OR:1.44,95%CI:1.09–1.90) were more likely to be non-compliant. Participants living further from VCs (OR:0.98,95%CI: 0.98–0.99) had better compliance. The major barriers were attitudinal (60.5%), economic (12.1%) and other medical or Health-related barriers (9.3%).

**Conclusion:**

The primary barriers to referral uptake were related to attitudes, economics, and medical. Participants not referred to teleophthalmology, unmarried, those without formal education, and those lived closer to VCs were more likely to be non-compliant. Addressing these barriers could improve the utilization of higher-level care services in this rural population.

## Introduction

An estimated 2.2 billion people worldwide experience near or distance vision impairment (VI), with at least 1 billion cases being potentially preventable or are currently unaddressed [1]. While cataract is the leading cause of blindness, uncorrected refractive error (URE) is the leading cause of VI [1]. Apart from this, there is a huge unmet need for presbyopia [2]. India is estimated to have around 34 million individuals living with blindness or moderate-to-severe visual impairment (MSVI) [3]. More than 90% of this is due to cataract and URE [4]. To address this challenge, the World Health Organization (WHO) had proposed universal eye health (UEH) using integrated people centred eye care (IPCEC) approach [5]. One way of achieving UEH is the vision centre (VC) approach, which involves activities related to the prevention and promotion, refraction and dispensing of spectacles, recognition of potentially blinding eye conditions, and appropriate referrals [6]. These VCs, aligned with the National Program for Control of Blindness (NPCB), provide essential eye care services in remote regions, thus improving access to eye care [7].

Access to eye care, is a multidimensional concept involving factors such as availability, affordability, approachability, acceptability, and appropriateness. The L V Prasad Eye Institute (LVPEI) addresses these dimensions through its pyramidal model of eye care. This model enables a scalable approach to eye care, allowing for most cases to be managed at the primary care level (VCs) and those requiring referral, are referred to secondary centres (SCs) [8]. Complex cases which cannot be managed at SC are referred to the tertiary centres (TC) or to the centre of excellence. Although more than 80–90% of eye conditions can be managed at the primary and secondary levels of care, many individuals do not make effective use of these services, including referral services [9]. The most reported barriers to the uptake of eye care services include financial constraints, transportation limitations, apprehension about surgical procedures, instances of unsatisfactory surgical outcomes observed in others, and a perceived lack of necessity [10–13]. Additionally, barriers in referrals from SC to TC include perceptions such as being able to see adequately, too busy to seek treatment, not recognizing vision loss as a serious issue, and accepting it as a natural part of aging [9,14,15].

However, we found no studies which has investigated the barriers to the uptake of referral services from the primary eye care level (VC) to the next higher level (SC). Therefore, this study aims to identify the barriers hindering the uptake of referral services from primary level VCs to higher level SCs.

## Methodology

The study was approved by the institutional ethics committee (Ethics Ref No LEC-BHR-R-09-21-745) of the Hyderabad Eye Research Foundation; L V Prasad Eye Institute, Hyderabad, India and conducted in accordance with the Declaration of Helsinki. This cross-sectional study was conducted using data from 10 VCs surrounding an SC. The SC, Kuchakulla Ramachandra Reddy Eye Centre (KRREC), is in Thoodukurthy village, Mahabubnagar district, Telangana, South India. The data was collected from the electronic medical records of participants referred to KRREC from 10 VCs. VCs provide basic eye care services, including history taking, visual acuity (VA), refraction, dispensing of spectacles, slit lamp examination, intraocular pressure measurement and identification of common ocular diseases, and referral to SC if needed. Referral is indicated for any participants whose VA does not improve to 6/18 or better for distance or N8 for near in either eye after spectacle correction, or who presents with any anterior or posterior segment abnormality. Referrals are categorized as routine or urgent. Routine referrals include conditions such as cycloplegic refraction, moderate cataracts, pterygium crossing the limbus, corneal scars, shallow anterior chamber, unexplained vision loss, and eyelid abnormalities like ptosis or blepharitis. Urgent referrals include sudden vision loss, ocular trauma, acute infections, cells and flare, red eye, high intraocular pressure, corneal infiltrates, neovascularization of iris, hypermature cataracts, leukocoria, relative afferent pupillary defect (RAPD), and suspected childhood vision problems [16].

Subsequently, we contacted these individuals via telephone to evaluate their compliance with treatment, and to understand the reasons for non-compliance among those referred for further treatment but failed to adhere to it. After this initial assessment we conducted home visits to administer a questionnaire aimed at identifying barriers to accessing referral services. The questionnaire, previously validated and utilized in our earlier study [9], was reused in this study by three trained field investigators. Written informed consent was obtained from all participants, before administering the questionnaire.

The study included participants who received primary eye care at VCs and were referred to an SC (KRREC) between July 1st to December 31st, 2019, and from July 1st to December 31st, 2020. The questionnaire was administered to these participants from 8^th^ November 2021–31st May 2023. Our intent was to capture both pre- and during COVID-19 impacts on referral and utilization of eye care services. Inclusion criteria included adults aged 18 years and older residing within a 50-kilometer radius of the SC, referred from a VC, with accessible contact information, and who was willing to provide informed consent. Exclusion criteria encompassed those below 18 years, residing beyond the 50-kilometer radius, those could not be contacted, and those referred to a general physician. Participants were classified as compliant if they attended the SC within one year of referral. Non-compliance was defined as failure to attend within this time frame. Non-compliant participants were interviewed regarding the reasons for non-adherence. Participants were considered unavailable if they were absent from their residence after three attempted home visits, scheduled at least one week apart. In addition to reasons for non-compliance, we also collected a range of data, including demographic details, distance from the SC in kilometres, referral status, presenting and best corrected distance VA, type of family, marital status, housing type, and level of education. Referral statuses were categorized as emergency or non-emergency based on the urgency and nature of the eye condition. Emergency referrals included participants with sudden vision loss, eye pain, redness, injuries, etc., while non-emergency referrals encompassed conditions that do not require immediate intervention, such as gradual vision deterioration. Family type was classified as nuclear (comprising one generation, i.e., parents and their children) or extended (including two or more generations, such as grandparents). Marital status was categorized as married, or unmarried/widowed. Housing type was divided into katcha and pucca. Pucca is permanent, well-constructed housing with durable materials and katcha is temporary or semi-permanent housing made of mud, thatch, or other fragile materials. Housing type serves as an indicator of socioeconomic status, with katcha housing generally associated with lower income levels and poorer living conditions. Educational level was classified as formal education (having completed at least primary education) or no formal education (lacking primary education). Participants were also categorized based on whether they were referred for teleophthalmology services or not. Eligibility for teleophthalmology services were based on the nature of the eye condition. Participants presenting with acute noticeable symptoms, such as sudden loss of vision, any obvious eyelid abnormality like chalazion, ptosis, dacryocystitis, meibomianitis blepharitis, stye, edema of lid, red eye (can be secondary to any ocular surface infection or inflammation), corneal and conjunctival pathologies such as epithelial defects, keratitis,corneal foreign body,opacities, limbitis,pterygium and lens related pathologies such as cataract, aphakia, posterior capsular opacification,uveitis and unexplained vision loss etc were prioritized for teleophthalmology service [17]. Among the total 1,507 participants, 207 (13.74%) were referred for teleophthalmology services. Barriers to referral uptake were categorized using a thematic analysis approach. Participant responses were assigned to predefined categories: economic, logistical, distance-related, fear-based, awareness-related, family-related, attitudinal, medical/ocular, and institutional barriers. Each response was classified into the most appropriate category based on its primary meaning.

### Statistical analysis

Data were meticulously collected and cross-checked for accuracy. To minimize data entry errors, double data entry was employed. Continuous variables were analysed using the student’s t-test, while categorical variables were assessed using either the chi-square test or Fisher’s exact test. Both univariable and multivariable logistic regression analyses were conducted to identify risk factors associated with non-compliance. Multicollinearity between variables was tested using variance inflation factors (VIF). Subsequently, the Hosmer-Lemeshow test for goodness-of-fit was applied to evaluate the model’s fit. A two-sided p-value of <0.05 was considered statistically significant. Data entry was carried out using Microsoft Access, and statistical analyses were performed using Stata 16.1 (StataCorp LLC, College Station, TX) for Windows.

## Results

Between July and December 2019 and July and December 2020, 2508, patients were referred to the SC. Among the 2508, 1930 (76.9%) were available for the study as participants. A total of 578 participants were excluded due to the following reasons: 191 participants were under 18 years of age, 2 participants were from outside the district, and 385 participants did not have contact numbers. Of the 1930 participants, 1507 (78%) interviewed. Among those, 938 (62.2%) were compliant, and 569 (37.8%) were non-compliant. The remaining 423 (22%) participants were unavailable. The reasons for their unavailability are as follows: 104 (24.6%) died, 12 (2.8%) migrated, 26 (6.1%) refused, 80 (19%) had wrong address, 41(9.7%) had wrong telephone number,104 (24.6%) did not respond to calls or had phone switched off, and 56 (13.2%) were not at home after three attempts.

The mean age of these participants was 54.64 years (SD: 14.28 years) and 47.5% were female, while the mean age of unavailable participants was 64.94 years (SD: 9.3), with 36.6% being female. Table 1 shows comparison of demographic variables, ocular characteristics and socioeconomic status between compliant and non-compliant groups. Non-compliance was significantly associated with distance from vision centres (p < 0.001), marital status (p = 0.04), and type of house (p = 0.02) (Table 1).

**Table 1:** Comparison of compliant and non-compliant groups by demographics, type of referrals, visual impairments, and socioeconomic status.

Factors	Complaint (N = 938)	Non-compliant (N = 569)	P value
	n (percentage)	n (percentage)	
**Age** (years) Mean ± SD	54.68 (14.49)	54.58 (13.93)	0.89
**Gender**			
Male	481 (51.28)	310 (54.48)	0.23
Female	457 (48.72)	259 (45.52)	
**Referred to teleophthalmology**			
Referred	140 (14.93)	67 (11.78)	0.09
Not referred	798 (85.07)	502 (88.22)	
**Distance from vision centres (kilometers)** Mean ± SD	45.16 (23.26)	38.42 (15.98)	<0.001
**Type of referral**			
Emergency	156 (16.63)	79 (13.88)	0.15
Non-emergency	782 (83.37)	490 (86.12)	
**Presenting visual acuity (PVA) in better eye**			
Normal (better than or equal to 6/18)	450 (48.18)	272 (47.97)	0.94
Any visual impairment (worse than 6/18)	484 (51.82)	295 (52.03)	
**Family head**			
Head of family	474 (50.53)	314 (55.18)	0.08
Not head of family	464 (49.47)	255 (44.82)	
**Marital Status**			
Married	698 (74.41)	396 (69.60)	0.04
Unmarried	240 (25.59)	173 (30.40)	
**Type of family**			
Nuclear	609 (64.93)	349 (61.34)	0.16
Extended	329 (35.07)	220 (38.66)	
**Type of house**			
Katcha	174 (18.55)	135 (23.73)	0.02
Pucca	764 (81.45)	434 (76.27)	
**Education**			
Formal education	295 (31.45)	162 (28.47)	0.22
No education	643 (68.55)	407 (71.53)	
**Main earning member**			
Primary wage earner	402 (42.86)	269 (47.28)	0.09
Not a primary wage earner	536 (57.14)	300 (52.72)	
**Medical insurance**			
Medical insurance present	783 (83.48)	482 (84.71)	0.53
Medical insurance not present	155 (16.52)	87 (15.29)	
**Monthly family income (rupees)**			
<16,000	453 (48.29)	257 (45.17)	0.24
≥16,000	485 (51.71)	312 (54.83)	
**COVID-19**			
Pre-COVID	442 (47.12)	261 (45.87)	0.64
Post-COVID	496 (52.88)	308 (54.13)	

Table 2 shows factors associated with non-compliance using univariable and multivariable analysis. Univariable analysis showed that participants residing closer to VCs, being unmarried, and living in a pucca house were significantly associated with non-compliance. Multivariable analysis indicated that not getting referred for teleophthalmology services (adjusted OR: 1.41, 95% CI: 1.00–1.99), unmarried status (adjusted OR: 1.32, 95% CI: 1.02–1.71), and lack of formal education (adjusted OR: 1.44, 95% CI: 1.09–1.90) remained significant predictors of non-compliance. Participants living further away from VCs (adjusted OR: 0.98, 95% CI: 0.98–0.99) had better compliance than those living closer to VCs.

**Table 2:** Risk factors for non-compliance (univariable and multivariable analysis).

Factors	Univariable OR (95% CI)	P-value	Multivariable Adjusted OR (95% CI)	P-value
**Age (Years)**	1.00 (0.99-1.01)	0.90	0.99 (0.98-1.00)	0.08
**Gender**				
Male	1.00		1.00	
Female	0.88 (0.71-1.08)	0.23	0.84 (0.63-1.12)	0.23
**Referred to teleophthalmology**				
Referred	1.00		1.00	
Not referred	1.31 (0.96-1.80)	0.09	1.41 (1.00-1.99)	0.05
**Mean distance from vision centres (kilometers)**	0.98 (0.98-0.99)	<0.001	0.98 (0.98-0.99)	<0.001
**Type of referral**				
Emergency	1.00		1.00	
Non-emergency	1.24 (0.92-1.66)	0.16	1.30 (0.91-1.86)	0.15
**Presenting visual acuity (PVA) in better eye**				
Normal (better than or equal to 6/18)	1.00		1.00	
Any visual impairment (worse than 6/18)	1.01 (0.82-1.24)	0.94	0.97 (0.77-1.23)	0.83
**Family head**				
Head of family	1.00		1.00	
Not head of family	0.83 (0.67-1.02)	0.08	0.86 (0.64-1.14)	0.30
**Marital Status**				
Married	1.00		1.00	
Unmarried	1.27 (1.01-1.60)	0.04	1.32 (1.02-1.71)	0.04
**Type of family**				
Nuclear	1.00		1.00	
Extended	1.17 (0.94-1.45)	0.16	1.20 (0.93-1.54)	0.15
**Type of house**				
Katcha	1.00		1.00	
Pucca	0.73 (0.57-0.94)	0.01	0.81 (0.62-1.06)	0.13
**Education**				
Formal education	1.00		1.00	
No education	1.15 (0.92-1.45)	0.22	1.44 (1.09-1.90)	0.01
**Main earning member**				
Primary wage earner	1.00		1.00	
Not a primary wage earner	0.84 (0.68-1.03)	0.09	0.85 (0.64-1.12)	0.25
**Medical insurance**				
Medical insurance present	1.00		1.00	
Medical insurance not present	0.91 (0.68-1.21)	0.53	0.95 (0.70-1.28)	0.73
**Monthly family income (rupees)**	0.99 (0.99-1.00)	0.68	0.99 (0.99-1.00)	0.48
COVID-19				
Pre-COVID	1.00		1.00	
During-COVID	1.05 (0.85-1.30)	0.64	1.05 (0.84-1.31)	0.66
Hosmer Lemeshow test				0.58

Table 3 shows the primary barriers to referral uptake identified in the non-compliant group. The major barriers to referral uptake, as reported by non-compliant participants, were attitudinal (60.5%), followed by economic (12.1%), and other medical or Health-related barriers (9.3%).

**Table 3:** The primary barriers preventing the non-compliant group from taking up referral services.

Categories	Major barriers	Numbers (%)
Economics	I cannot afford to travel cost to the centre	4(0.70)
	I cannot afford the treatment costs	65(11.42)
	Cannot afford lost wages of me or accompanying person	0(0)
Logistics	There is nobody to accompany me to the secondary centre	29(5.10)
	I do not know where the secondary centre is located	1(0.18)
Distance	The secondary centre is very far from my home	6(1.05)
	Lack of transport	0(0)
Fear	I am afraid of traveling to the secondary centre	6(1.05)
	I am afraid of the procedure for which I have been referred for	12(2.11)
	Fear of COVID-19	10(1.75)
Awareness	I do not understand why I need to go a secondary centre	3(0.53)
	I was not aware of referral	9(1.58)
Family	The dominant family member does not feel the need for further travel and treatment	4(0.70)
Attitude	I am too busy to go to the eye centre for further treatment	137(24.08)
	Can manage now and will go later	44(7.73)
	I am happy with the treatment at VC and do not require further treatment at this time	163(28.64)
Medical or Health-related barriers	I was informed that my vision would not improve	1(0.18)
	Other health problems prevent me from traveling to the eye centre	37(6.50)
	Old age – I do not see the need for treatment at my age	15(2.64)
Institutional barriers	L V Prasad did not help to arrange the appointment and facilitate the referral	5(0.88)
	I am not satisfied with the treatment I have received thus far from L V Prasad	8(1.41)
	I decided to visit another eye centre/ visited other centre	5(0.88)
Others^*^	Others	5(0.87)

* Others: Spouse illness: 4(80%), Waiting for husband’s eye surgery: 1 (20%).

## Discussion

This is the first study that assesses the barriers to non-compliance with the uptake of referral services from VCs to SC within a major eye care network (LVPEI). Our study observed that approximately 38% of referrals were non-compliant. The primary barriers for non-compliance were “I am happy with the treatment at VC and do not require further treatment at this time” and “I am too busy to go to the eye centre for further treatment.” These barriers are consistent with previous studies [15,18]. This could be because participants felt that their current treatment is managing their condition effectively leading to complacency and a lack of urgency to seek further care. They might also assume that their condition will not worsen significantly if they delay care. Patients might not be aware about the importance of follow-up care and the potential consequences of non-compliance. Awareness about the condition, treatment plan, and the benefit of ongoing care is crucial to ensuring that participants understand the need for and the value of continuing treatment. Prima Moinul et al. found that compliance with follow-up care among patients with diabetic retinopathy improved by over 15% following an educational program that detailed the impact of diabetes on eye health, potential causes of vision loss, treatment options, risk factors, and the necessity of regular annual screenings [19]. Another explanation could be their busy work schedules, which can make it difficult to prioritise eye care. This is particularly true if taking time off work or managing other responsibilities poses a significant challenge.

The third-most common barrier was “I cannot afford the treatment costs”. Economic constraints have been identified as a primary obstacle to eye care utilisation in previous studies [9,20]. We have reported that financial difficulties hindered16.4% of patients from seeking further care (from SC to TC) [15]. Income levels substantially impact the affordability of eye care services. Both direct costs, such as cost of treatment, medications, and spectacle expenses, and indirect costs, such as lost wages for patients and caregivers, contribute to this challenge [21,22]. In certain situations, this could be attributed to the need to prioritize basic necessities like earning money for food, which takes precedence over addressing eye health issues due to opportunity costs [23]. On the other hand, the LVPEI model makes eye care accessible to numerous patients at little to no cost. This suggests a lack of patient awareness regarding available financial assistance. Enhancing financial counselling and promoting awareness of these services at the point of referral could address this issue.

Participants not referred for teleophthalmology services exhibited a significantly higher non-compliance rate (38% vs. 32%). While this could be attributed to the clinical profile of referred cases often involving anterior segment diseases with acute, noticeable symptoms. Our multivariable analysis did not find a statistically significant association between emergency referral status and compliance (p = 0.15). This suggests that clinical urgency may not be the key driver of improved follow-up. What stood out in our findings was the dual level of examination and care involved in teleophthalmology referrals. These participants underwent assessment both by the vision technician and by an ophthalmologist, which may have reinforced the importance of the diagnosis and the need for follow-up. This structured approach likely enhanced patient understanding and trust, contributing to higher compliance rates.

Our study showed that participants living closer to VCs are less likely to take up referral services. This could be because LVPEI provides transportation for travel to and from the SC, for patients who stay far away from SC whereas this facility is not available to VCs closer to SC. The easy availability of transportation made it easier for patients from faraway VCs to reach the SC. In contrast, patients from nearby VCs do not receive such transportation, possibly assuming that since the SC is closer, they can make their own way there. However, without organized transportation, it seems some patients may face difficulties in traveling, leading to fewer referrals attending SC.

We also found that unmarried participants were less likely to take up referral services. This is consistent with a prior study. Being unmarried is associated with a higher risk of poor health follow-up, with this risk being more pronounced for women, as opposed to men [24]. Unmarried individuals also might lack immediate social support as opposed to married individuals. Also, unmarried individuals might face different financial pressures compared to their married counterparts [25]. This was also seen from our data where the average income of unmarried/widowed participants was significantly lower than married participants (average monthly income INR 14,420 vs INR 17,605; p < 0.001). However, a PolSenior study with a 10-year follow-up found that single elderly individuals were more likely to undergo cataract surgery [26]. This contrast could be due to differences in healthcare systems, cultural attitudes toward independence, or variations in the availability of family support in different populations. More research is needed to explore how marital status influences healthcare utilization across diverse settings.

Participants with no formal education were less likely to take up referral services. This finding is similar to another study on three rural districts in southern India [13]. Individuals without formal education might have limited knowledge about available referral services and how to access them. Education often provides crucial information about health resources and how to navigate the healthcare system. Less education is often associated with reduced health literacy. This results in difficulties in understanding medical information, instructions, and the importance of seeking referral services, which can affect their decision to engage with these services [27]. Addressing these barriers may involve improving health education, increasing awareness of available services, and providing additional support to help individuals with no formal education navigate the healthcare system.

The primary strength of this study lies in its novelty, being the first study to examine non-compliance with referrals from primary care to a higher level of care within a rural setting. This research elucidates the key factors contributing to the low uptake of referral services and identifies specific groups more prone to non-compliance. However, a limitation of this study is that it was conducted within a distinct healthcare network in Southern India, which may limit the generalizability of the findings to other settings. The study population reflects the typical demographic and socioeconomic profile of rural South Indian communities, with a high proportion of individuals over 50 years of age, low levels of formal education, and moderate-to-low household income. These characteristics are broadly comparable to populations served by other rural eye care programs in India, enhancing the relevance of our findings to similar low and middle-income countries (LMIC) contexts. While we did not conduct a formal comparison with other regions, the demographic characteristics of our study population such as age, income, and education could broadly consistent with those seen in other rural Indian eye care populations. The unavailability of a substantial proportion of participants may have introduced selection bias, as their demographic characteristics differed from those who were available, potentially influencing the representativeness of the sample. Furthermore, due to limited sample size, we did not link primary barriers with non-compliant barriers. Additionally, our study focused on quantitative assessment of referral barriers and did not employ in-depth qualitative methods, such as the Theoretical Domains Framework (TDF). Future research should explore patient attitudes and behavioral barriers using qualitative approaches for deeper insights. The study also did not assess potential confounders or effect modifiers, and barriers to referral uptake could not be analyzed by individual diagnosis due to the limited sample size. Moreover, due to the relatively small number of referrals per VC, we did not analyze referral uptake on a VC-specific basis.

In conclusion, this study is the first to examine non-compliance with referrals from VCs to SC within our network, revealing a 38% rate of non-compliance. Key barriers to referral uptake included attitudinal and financial hardships. Also, our study highlights that participants living closer to VCs, unmarried participants, and those without formal education were also more likely to be non-compliant. Future strategies should focus on comprehensive approaches that include patient education, social support, improved access to financial support, and effective logistical arrangements to bridge the gap in referral uptake. Also, teleophthalmology should be encouraged at primary level which can additionally improve referral uptake. By addressing these multifaceted challenges, we can improve compliance rates and ultimately enhance patient outcomes in ophthalmologic care.
